# Performance of adenosine deaminase in detecting paediatric pleural tuberculosis: a systematic review and meta-analysis

**DOI:** 10.1080/07853890.2022.2140452

**Published:** 2022-11-08

**Authors:** Feiyang Na, Yannan Wang, Hui Yang, Li Guo, Xuan Liang, Donghai Liu, Rongfang Zhang

**Affiliations:** a Department of Allergy, Gansu Provincial Maternity and Child-care Hospital, Lanzhou, China; b Department of Radiology, Lanzhou University Second Hospital, Lanzhou, China

**Keywords:** Adenosine deaminase, paediatric pleural tuberculosis, diagnose, sensitivity, specificity

## Abstract

**Background:**

Paediatric pleural tuberculosis (TB) is a paucibacillary disease, which increases the difficulty of examination. We aimed to assess the performance of pleural fluid adenosine deaminase (ADA) in the detection of paediatric pleural TB.

**Methods:**

PubMed, Web of Science Core Collection, Embase and Cochrane Library databases were searched up to 20 December 2021. We used the bivariate and hierarchical summary receiver operating characteristic models to compute pooled estimates for the overall diagnostic accuracy parameters of ADA for diagnosing paediatric pleural TB.

**Results:**

Eight studies, including 290 pleural fluid samples, met the inclusion criteria. The pooled sensitivity of ADA was 0.85 (95% CI: 0.78–0.90, I^2^: 55.63% < 75%) for detecting patients with paediatric pleural TB. A total of 262 pleural fluid samples from four studies were included to differentiate patients with paediatric pleural TB from controls. At a unified cut-off value of 40 U/L, the pooled sensitivity, specificity, positive likelihood ratio, negative likelihood ratio, diagnostic odds ratio and area under the summary receiver operating characteristic curve of ADA were 0.89, 0.58, 2.09, 0.20, 10.48 and 0.89, respectively.

**Conclusions:**

At a cut-off value of 40 U/L, the overall performance of ADA was good for detecting paediatric pleural TB, with relatively high sensitivity and low specificity.

**Key message**
- Accurate identification of paediatric pleural TB will help eliminate TB in children. At a cut-off value of 40 U/L, the overall performance of ADA was good for detecting paediatric pleural TB, with relatively high sensitivity and low specificity.

## Background

Paediatric tuberculosis (TB) differs from TB among adults, wherein children have a higher risk of progression to active TB [1]. In 2019, an estimated 10 million individuals developed TB worldwide, as reported by the World Health Organization (WHO) [2]. Of these, 12% were paediatric patients aged under 15 years despite Calmette–Guerin bacillus vaccine protection [2]. Pleural TB, which is a common form of extrapulmonary tuberculosis, occurs in approximately 4–15% of paediatric TB cases [3–4]. Paediatric pleural TB is paucibacillary, which increases the difficulty in examination [5], resulting in delayed treatment. Thus, more diagnostic aid to assess paediatric pleural TB and initiate early treatment are necessary to eliminate TB.

In hospitals, the clinical manifestations of paediatric pleural TB are nonspecific. The standard diagnosis for paediatric pleural TB is positive results for mycobacterial cultures, which take a relatively long time (6–12 weeks) and show negative results in 70% of cases [6,7]. Because of the low bacterial load in children, the positivity for acid-fast bacilli (AFB, 5.4%) and on polymerase chain reaction (PCR, 14.3%) is low [6,8]. Pleural biopsy has a better diagnostic performance for detecting pleural TB; however, it is invasive [9,10]. Consequently, finding some pleural fluid biomarkers could be a simple, quick and non-invasive auxiliary detection method.

Adenosine deaminase (ADA) is an enzyme mainly produced by lymphocytes, and it participates in the metabolism of purines [11]. Pleural fluid ADA levels increase significantly in adults with pleural TB. Using the most commonly used cut-off value of 40 U/L, many studies (including systematic review and meta-analysis) have indicated that pleural fluid ADA has a high sensitivity (0.92–0.93) and specificity (0.90–0.92) among adults [8,11–14]. Moreover, ADA detection is fast, inexpensive and minimally invasive [15]. However, until now, the diagnostic accuracy of ADA at a cut-off value of 40 U/L in paediatric pleural TB remains unclear [16–22]. Thus, this systematic review and meta-analysis was aimed at evaluating the diagnostic performance of ADA in the detection of paediatric pleural TB.

## Methods

### Search strategy

This study was performed according to the Preferred Reporting Items for Systematic Reviews and Meta-Analyses Diagnostic Test Accuracy statement published in 2018 [23]. The protocol for this systematic review and meta-analysis was available online (PROSPERO: CRD 42021275605). Approval from the institutional ethics committee was not required because only published articles were included.

We searched the PubMed, Web of Science Core Collection, Embase and Cochrane Library databases for English publications from their inception dates to 20 December 2021. The search terms for all fields were as follows: (tuberculosis OR tuberculous OR mycobacterium tuberculosis OR tubercular OR TB OR MTB) AND (pleural OR pleura OR pleuritis OR pleurisy OR extrapulmonary) AND (child OR children OR paediatric OR paediatric OR infant OR newborn OR neonate OR toddler OR adolescent) AND (adenosine deaminase OR ADA). Besides, we identified the bibliographies and records of the selected publications to check possibly eligible articles.

### Selection criteria

Studies reporting the use of ADA for detecting paediatric pleural TB were selected on the basis of the following criteria: (1) participants included paediatric pleural TB patients and/or non-paediatric pleural TB controls (more than five participants); (2) detection of ADA in pleural fluid was the index test, with a united cut-off value of 40 U/L; (3) one or more diagnostic standards were selected, including tuberculosis culture, AFB, PCR, and pleural biopsy, for determination of histopathological and clinical characteristics; (4) sensitivity and/or specificity of ADA were the major outcomes and (5) the studies were randomized controlled trials or observational trials (cohort and cross-sectional studies). When more than one ADA cut-off value was used, we selected the most used cut-off value of 40 U/L for data analysis [11,15].

We excluded animal experiments, non-English publications, case reports, reviews, guidelines and recommendations, articles not reporting paediatric pleural TB and studies not assessing pleural fluid ADA. Two investigators independently screened the titles, abstracts and full texts to meet the inclusion criteria, and disagreements were resolved by discussion.

### Data extraction

Two reviewers extracted data independently from the eligible articles, namely, the first author, year of publication, TB burden condition (high or low), study design (retrospective or not), number of participants (patients and controls), types of controls (yes or no, if yes, which types of controls), diagnostic reference standard for paediatric pleural TB, index test (ADA, IU/L), source of samples, ages, most commonly used cut-off value of ADA, sensitivity and/or specificity, true positive (TP) results, false positive (FP) results, false negative (FN) results and true negative (TN) results. Data that only provided information on the sensitivity of ADA for detecting paediatric pleural TB in patients were extracted for this study.

### Quality assessment

The quality assessment of diagnostic accuracy studies-2 (QUADAS-2) tool was used to describe and summarize the methodological quality of the included studies using RevMan software (version 5.3; Cochrane, London, UK) [24]. It consists of four domains: patient selection, index test, reference standard and flow and timing.

### Statistical analysis

We used a bivariate random model to compute the pooled sensitivity, specificity, positive likelihood ratio (PLR), negative likelihood ratio (NLR), diagnostic odds ratio (DOR), and area under the summary receiver operating characteristic curve (AUC) for ADA in detecting paediatric pleural TB, with corresponding 95% confidence intervals (CI). Additionally, we constructed a hierarchical summary receiver operating characteristic (HSROC) plot to summarize the detection performance of ADA among paediatric participants [25]. We considered heterogeneity to be present if the I^2^ statistic was >75% [26]. The Galbraith plot was used to explore the heterogeneity, and the outlier studies which could be found in this plot showed possible heterogeneity. According to the general expert advice, we used the most common diagnostic threshold (cut-off: 40 IU/L) of ADA for the data analysis [11,15]. Other cut-off values were excluded. The possible existence of publication bias was assessed for using Deek’s funnel plot, and a *p*-value < 0.10 indicated a possible bias [27].

The statistical software Stata (version 14.0; StataCorp, College Station, TX, USA) was used to analyze the data, with ‘midas’, ‘metandi’ command packages.

## Results

### Studies retrieved and their characteristics

**Figure 1.**
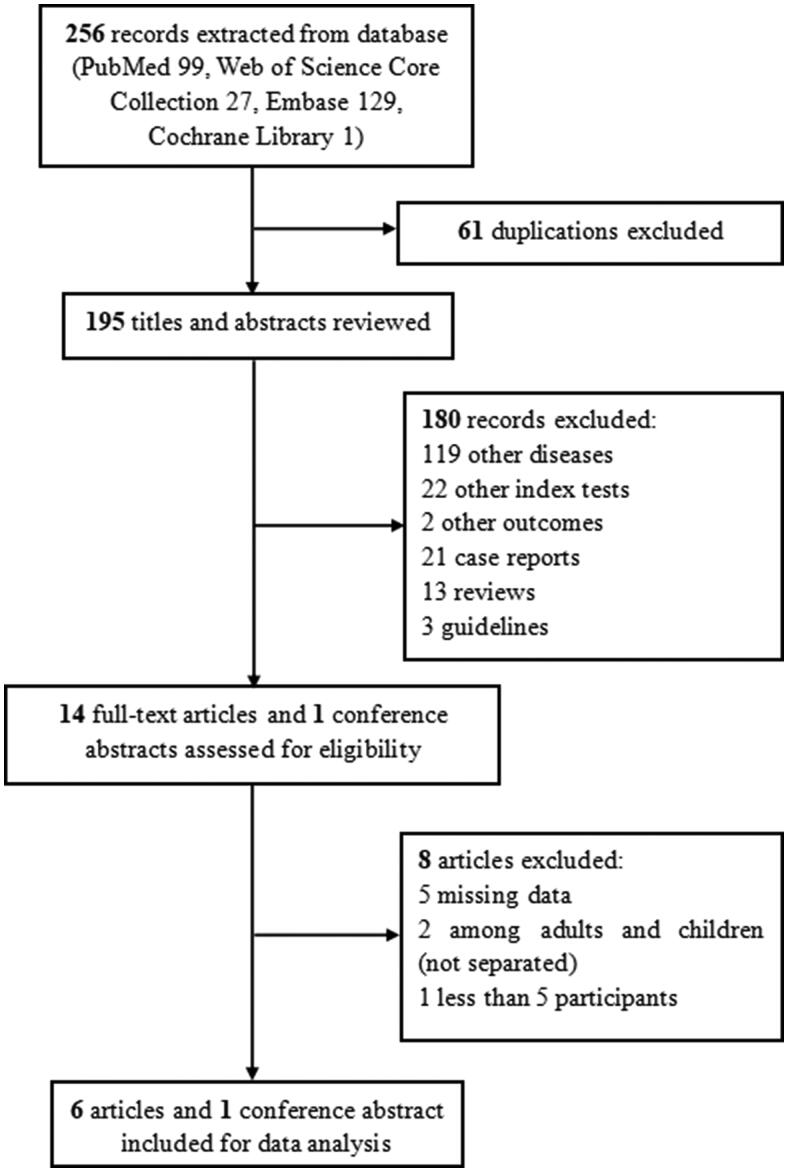
The selection process.

A total of 256 records that potentially met the inclusion criteria were retrieved from four databases. We excluded 61 duplications. By reviewing the titles and abstracts, 180 records were not eligible, including records of other diseases (119 records of pulmonary tuberculosis among adults, tuberculous meningitis, tuberculous pericardial effusion, etc.), with other index tests (22 records of Xpert MTB/RIF Ultra, pleural fluid C-reactive protein, and interferon-gamma release assays, etc.), with other outcomes (two records), case reports (21 records), reviews (13 records) and guidelines (3 records). Fourteen full-text articles and one conference abstract were assessed. Finally, we included six articles and one conference abstract for data analysis (Figure 1).

Detailed characteristics of the seven publications (eight studies) are presented in Table 1. The year of publication ranged from 1999 to 2021. Five studies (62.5%) were from high TB burden countries [16–18,21] and three studies (37.5%) were from low TB burden countries [19,20,22]. Eight studies (100%) were retrospective study designs [16–22]. The eligible studies included 290 paediatric patients with pleural TB and 157 paediatric controls. Only four studies had controls, of which one study focussed on parapneumonic effusion [16], one study focussed on parapneumonic effusion (excluding purulent pleural effusion) [16], one study did not describe the details [17] and one study focussed on empyema thoracis and malignant pleural effusion [18]. All reference standards for paediatric pleural TB were composites. The level of ADA in the pleural fluid was extensive. The ages of the participants ranged from 0 to 18 years. We used a unified cut-off value of 40 U/L of ADA in these individual studies. The sensitivity, specificity, TP, FP, FN and TN values are shown in Table 2.

**Table 1.** Characteristics of included studies.

Author	Year	Country	TB burden	Study design	Participants(N)		Controls	Reference standard	Index test (ADA, U/L)		Sample
					TPE	Non-TPE			TPE	Non-TPE	
Vieira et al. [16]	2021	Brazil	High	Retrospectively	25	68	Parapneumonic effusion	Composite	159.7 ± 66.3	117.6 ± 107.5	Pleural fluid
Vieira et al. [16]	2021	Brazil	High	Retrospectively	25	59	Parapneumonic effusion (excluding purulent pleural effusion)	Composite	Unknow	Unknow	Pleural fluid
Bhutia et al. [17]	2011	India	High	Unknow	35	19	Non tubercular pleural effusion	Composite	114.74 ± 61.25	30.89 ± 6.66	Pleural fluid
Mishra et al. [18]	2006	India	High	Unknow	20	11	Empyema thoracis and malignant pleural	Composite	Unknow	Unknow	Pleural fluid
Lupu et al. [19]	2020	Moldova	Low	Retrospectively	56	None	None	Composite	Unknow	None	Pleural fluid
Bayhan et al. [20]	2018	Turkey	Low	Retrospectively	7	None	None	Composite	50.8 ± 12.3	None	Pleural fluid
Wang et al. [21]	2015	China	High	Retrospectively	102	None	None	Composite	58.7 ± 31.8	None	Pleural fluid
Merino et al. [22]	1999	Spain	Low	Retrospectively	20	None	None	Composite	73.8 ± 11.9	None	Pleural fluid

Abbreviations: TB: tuberculosis; TPE: tuberculous pleural effusion; ADA: adenosine deaminase.

**Table 2.** Detecting performance of ADA for all the included articles.

Author	Year	Age (year)		Cut-off (ADA)	Sensitivity	Specificity	TP	FP	FN	TN
		TPE	Non-TPE							
Vieira et al. [16]	2021	13.4 (7.4–14.7)	5.4 (2.4–13.9)	40 U/L	0.88	0.31	22	47	3	21
Vieira et al. [16]	2021	0–18	0–18	40 U/L	0.88	0.36	22	38	3	21
Bhutia et al. [17]	2011	5.14	6.43	40 U/L	0.94	0.89	33	2	2	17
Mishra et al. [18]	2006	2–14	2–14	40 U/L	0.8	0.73	16	3	4	8
Lupu et al. [19]	2020	0–18	None	40 U/L	0.84	None	47	None	9	None
Bayhan et al. [20]	2018	14 ± 1.4	None	40 U/L	0.857	None	6	None	1	None
Wang et al. [21]	2015	11.6 ± 3.2	None	40 U/L	0.745	None	76	None	26	None
Merino et al. [22]	1999	0–18	None	40 U/L	0.9	None	18	None	2	None

Abbreviations: TPE: tuberculous pleural effusion; ADA: adenosine deaminase; TP: true positive; FP: false positive; FN: false negative; TN: true negative.

### Risk-of-bias assessments

**Figure 2.**
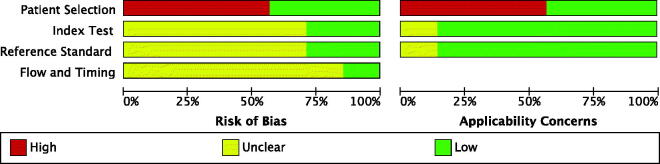
Quality for the eligible articles.

An assessment of the risk of bias is shown in Figure 2. According to the QUADAS-2 tool, we observed an unclear risk of bias for the study that only had an abstract [19], a high risk of bias for studies that only provided data on the sensitivity of ADA [20–22], and a low risk of bias for the rest of the studies [16–18]. In terms of the patient selection domain, four studies (57.1%) without controls were high risk [19–22]. For the index test and reference standard domains, investigators did not provide clear information on study blinding in five studies (71.4%) [16,17,19–21]. In the flow and timing domains, six studies (75%) were unclear because of missing participants in the data analysis [16,18–22].

### Pooled analysis of ADA among children

A total of 290 pleural fluid samples from eight studies using ADA were evaluated for sensitivity of paediatric pleural TB. The sensitivity ranged from 0.75 to 0.94, with a pooled sensitivity of 0.85 (95% CI: 0.78–0.90, I^2^: 55.63% < 75%).

**Figure 3.**
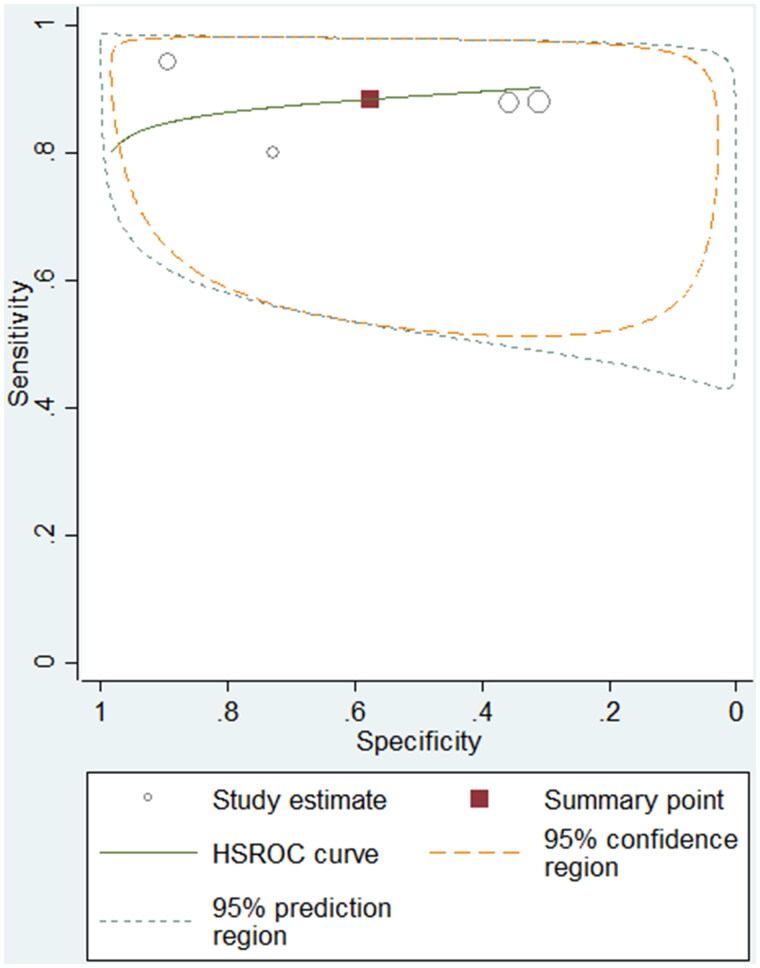
Hierarchical summary receiver operating characteristic (HSROC) plot to summarize diagnostic accuracy for ADA in detecting paediatric pleural TB.

Besides, we included 262 pleural fluid samples from four studies to differentiate patients with paediatric pleural TB from controls. The sensitivity differed from 0.80 to 0.94, and the pooled sensitivity was 0.89 (95% CI: 0.80–0.94, I^2^: 3.14% < 75%). The specificity varied from 0.31 to 0.89, and the pooled specificity was 0.58 (95% CI: 0.29–0.82, I^2^: 90.2% > 75%). The pooled PLR and NLR were 2.09 (95% CI: 1.01–4.30) and 0.20 (95% CI: 0.08–0.47), respectively. The summary DOR was 10.48 (95% CI: 2.30–47.68), and the AUC was 0.89 (95% CI: 0.86–0.91). The HSROC curve for ADA is shown in Figure 3. The Galbraith plot identified no outlier studies, which suggested no heterogeneity within the included studies.

### Publication bias

No publication bias was observed on Deeks’ funnel plots, and no statistically significant differences in ADA (*p* = 0.55) existed between paediatric patients with pleural TB and controls.

## Discussion

Our systematic review and meta-analysis emphasized the diagnostic value of ADA in paediatric patients with pleural TB. This study showed that ADA had a good sensitivity (0.89) for detecting paediatric pleural TB at the unified and most commonly used cut-off value of 40 U/L; however, the specificity (0.58) was not good for distinguishing paediatric controls. The AUC was 0.89, indicating that the overall diagnostic performance of ADA was good for differentiating paediatric pleural TB.

Previous meta-analyses suggested that pleural fluid ADA had a good diagnostic performance for pleural TB among adults [11,28]. In 2003, Goto et al. first evaluated the detection value of pleural fluid ADA and found it to be reasonably good (92.2% for both sensitivity and specificity) for pleural TB among adults, with 40 articles included [14]. In 2008, Liang et al. included 63 studies and obtained similar results [13]. In 2019, Aggarwal et al. updated the summary sensitivity (0.92) and specificity (0.90) of ADA and found it to be good for detecting pleural TB among adults, including 174 publications (2 publications were children) with 27,009 participants [11]. When differentiating between different TB burden regions, Aggarwal et al. focussed on a high TB burden country (India) in 2016, indicating that ADA had a good sensitivity (0.94) and specificity (0.89) [28]. Palma et al. concentrated on Spain (low TB burden) in 2019, suggesting that ADA still had a good sensitivity (0.93) and specificity (0.92) [12]. These meta-analyses were more concerned with adults. The ADA has been confirmed as a good diagnostic method among adults; however, whether it has the same results in children has never been studied. Our study showed a difference in specificity between adults (0.90) and children (0.58).

The screening test for detecting tuberculosis in children has minimal sensitivity (90%) and specificity (70%), as recommended by the WHO [29]. The sensitivity in our study was similar to the recommended sensitivity (0.89 and 0.90). However, the specificity was much lower than the recommended specificity (0.58 vs 0.70). There are several possible reasons for this. On the one hand, the most prevalent aetiologies of pleural effusion in children are TB and pneumonia, and parapneumonic effusion and empyema shows frequently high ADA level. In our study, controls included parapneumonic effusion and empyema participants, which resulted in elevated ADA levels for the controls. In clinic, cell counts could be further used for differential diagnosis, in which parapneumonic effusion and empyema are neutrophilic, and tuberculous pleural effusion lymphocytic. On the other hand, we only included one cut-off value of 40 U/L and the specificity was lower than the optimal cut-off value of 125 U/L (0.36 vs 0.73) [16]. The small number of eligible studies may limit the reliability of this result, as well.

The present study has some limitations. First, the retrospective study design and the small sample size of the included studies led to wide CIs. Although pleural TB is a common extrapulmonary TB, paediatric pleural TB is less common. Second, all studies used composite criteria for diagnosing paediatric pleural TB, and thus misclassification bias could not be ruled out. Meanwhile, selection bias wouldn’t be ignored. Third, even though Galbraith plot indicated no heterogeneity, there still had potential heterogeneity existing due to the sparsity of the included studies. Moreover, the controls in these studies were potentially different. Mishra et al. included some malignant PE controls, with a relatively high specificity (0.89) [18]. In clinic, ADA levels are known to be considerably lower in participants with malignant PE than in those with tuberculous PE, which might be an explanation for the different specificities. Further studies could pay more attention to controls with different population, including malignant PE, parapneumonic effusion, etc. Additionally, no publication bias existed in this study according to Deeks’ funnel plots, which was limited by the small number of eligible studies. Finally, the fact that only English articles were included may have led to research in other languages being missed.

## Conclusions

In conclusion, at a cut-off value of 40 U/L, our systematic review and meta-analysis showed that the overall performance of ADA is good for detecting paediatric pleural TB. The sensitivity of ADA is good for detecting paediatric patients with pleural TB, whereas the specificity is not good for distinguishing paediatric controls. Further large-sample and prospective studies on paediatric pleural TB are needed to determine whether our findings are correct.

## Data Availability

All data are available.
